# Psychometric validation of diabetes distress scale in Bangladeshi population

**DOI:** 10.1038/s41598-021-04671-0

**Published:** 2022-01-12

**Authors:** Jesmin Akter, Rakibul M. Islam, Hasina Akhter Chowdhury, Shahjada Selim, Animesh Biswas, Tanvir Ahmed Mozumder, Jonathan Broder, Dragan Ilic, Md Nazmul Karim

**Affiliations:** 1 Bangladesh Center for Communication Programs, Dhaka, Bangladesh; 2 School of Public Health and Preventive Medicine, Monash University, Melbourne, Australia; 3 Department of Biostatistics, Bangladesh University of Health Sciences, Dhaka, Bangladesh; 4 Department of Endocrinology & Metabolism, Bangabandhu Sheikh Mujib Medical University, Dhaka, Bangladesh; 5 BRAC University, Dhaka, Bangladesh

**Keywords:** Diseases, Endocrinology, Health care

## Abstract

Diabetes Distress (DD)—an emotional or affective state arise from challenge of living with diabetes and the burden of self-care—negatively impact diabetes management and quality of life of T2DM patients. Early detection and management of DD is key to efficient T2DM management. The study aimed at developing a valid and reliable instrument for Bangladeshi patients as unavailability such a tool posing challenge in diabetes care. Linguistically adapted, widely used, 17-item Diabetes Distress Scale (DDS), developed through forward–backward translation from English to Bengali, was administered on 1184 T2DM patients, from four diabetes hospitals in Bangladesh. Psychometric assessment of the instrument included, construct validity using principal component factor analysis, internal consistency using Cronbach’s α and discriminative validity through independent t-test and test–retest reliability using intraclass-correlation coefficient (ICC) and Kappa statistics. Factor analysis extracted 4 components similar to original DDS domains, confirms the construct validity. The scale demonstrated satisfactory internal consistency (α = 0.838), stability (test–retest ICC = 0.941) and good agreement across repeated measurements (Kappa = 0.584). Discriminative validity revealed that patients with complication (p < 0.001) and those are on insulin (p < 0.001) had significantly higher distress scores in all domains. Bengali version of DDS is a valid and reliable tool for assessing distress among Bangladeshi T2DM patients.

## Introduction

Diabetes mellitus (DM) has emerged as a global pandemic, becoming a major global health challenge over the past few decades^1,2^. As of 2019, around 463 million adults (aged between 20 to 79 years) were suffering from DM, of which 79% were from low-and middle-income countries^3^. These figures are expected to increase to 700 million by 2045, if current trend of increase continues^3^.

Living with DM can be debilitating and challenging due to its physical and psychological impact on individual’s health and wellbeing as well as the distress specific to diabetes, its complications and management^4–6^. Diabetes distress (DD) refers to the distinctive, primarily emotional, worries and burdens that are facets of the spectrum of patient experience in-course of managing and living with diabetes^7^. DD is more of a negative emotional state, a rational response to the arduous set of self-care behaviors and is not considered a psychopathology^8^ unless start to affect patient’s day-to-day activities, diabetes management and also interpersonal relationships. Symptoms of DD are similar to those of depressive disorder, however they are not severe enough to meet the diagnostic criteria for major depressive disorder^9,10^. Patients with T2DM may exhibit symptoms of defeat, denial, fear, isolation, frustration and poor motivation^11^. DD can be distinguished from clinical depression in its nexus with management of the disease, glycemic control in particular^12,13^. Unmitigated condition is likely to lead to “burnout”—an emotionally overwhelmed and exhausted state that may prevent people with T2DM from achieving optimal glycemic control^7^.

With the continued rise of diabetes burden, DD is projected to become a major concern for people living with DM in the current century^5,6^. DD have been linked with a range of outcomes including poor self-care, low diabetes self-efficacy, and poor quality-of-life (and subsequently poor glycemic control), earlier onset of complications, frequent admissions to hospital care, and increased mortality^14–16^. Diabetes management strategies consider patients emotional state as a key element^17^. Failure to mitigate potential distress in patients may impede their implementation^18,19^. Hence periodic assessment of DD should be enshrined in the management protocols of DM^13,17^. There are a handful of instruments available for assessing diabetes distress^20–24^, of which the Diabetes Distress Scale (DDS) is considered most efficient among the most widely used globally^24^.

The number of people with type 2 DM (T2DM) in Bangladesh is projected to be 13.6 million by 2040 compared with 6.9 million in 2017^25^, and is likely to incur substantial health and economic burden^26,27^. Although an earlier study^28^ in Bangladesh studied the prevalence of DD in diabetic population, however they used an unvalidated tool due to absence of a psychometrically validated in Bangladeshi population. While, generic tools can be used for diagnosing T2DM related distress, a DM-specific and psychometrically validated instrument may increase the precision of detection and thus help in choice of most appropriate intervention thereof. The primary goal of the study hence is to develop a linguistically validated DD detection tool for Bangladeshi patient population which can be used clinical and research setting. The current study, thus aimed to translate the DDS-17 in Bengali and to perform psychometric validation in Bangladeshi T2DM patient population.

## Methods

### Patients recruitment

Adult (≥ 18 years) having T2DM for at least one year, who had been attending the study hospitals for diabetes care between October 2018 to March 2019, were approached at the end of the routine visit with a health professional, and were informed verbally about the purpose and protocols of the study and the time required for their participation. Those consented were included in the study. The study was conducted in four tertiary care hospitals providing diabetic care, from Dhaka district—Bangladesh Institute of Health Sciences General Hospital, Bangabandhu Sheikh Mujib Medical University and Universal Medical College Hospital- and from Kushtia district—Bheramara Diabetic Shomity Hospital. These facilities serve both rural and metropolitan catchment areas and thus serve diabetic population of diverse sociodemographic and economic strata.

### Sample size determination

Using the current prevalence of T2DM of 6.9%^3^, with 95% confidence interval with a 2.5% margin of error, the minimum sample size required was 1180 for effective analysis in subgroups such as sex (men/women) and place of residence (metropolitan/non-metropolitan).

### Data collection

Face to face interviews were conducted by trained interviewers. At the end of the interview, patients were asked if they are interested to participate in a follow-up interview in a further 4-weeks’ time and a second appointment was scheduled for those who consented. Patients were excluded from the study if they were pregnant or nursing, had a diagnosis of dementia or psychosis, or had severe debilitating comorbidities (e.g. stroke, cancer). Prior to analysis, data have been screened for discrepancy and completeness, where possible Incomplete records have been completed from the medical records or where applicable in communication with the patient. Participants with incomplete records were excluded from the analysis (complete case analysis). The study was approved by the Bangladesh University of Health Sciences (BUHS) Ethical Review Committee, Dhaka, Bangladesh (Approval No. BUHS/BIO/EA/18/10). Informed written consent was obtained from all participants prior to the inclusion in the study. The study was conducted in accordance with the relevant methodological guidelines and regulations.

### Diabetes distress scale

DDS17 is developed by Polonsky et al.^24^ for the assessment of diabetes related distress. The scale consists of 17 items and four subscales: (i) emotional burden EB (five items: 1, 3, 8, 11, 14), (ii) physician-related distress PD (four items: 2, 4, 9, 15), (iii) regimen-related distress RB (five items: 5, 6, 10, 12, 16) and (iv) diabetes-related interpersonal distress ID (three items: 7, 13, 17). The scale reported to have a consistent, generalizable factor structure and good internal consistency reliability (Cronbach’s α) of the main scale (DDS17 = 0.87) and the four subscales (EB = 0.88, PD = 0.88, RD = 0.90, and ID = 0.88) were adequate. Original DDS was developed in English and subsequently been translated in over 10 languages including Danish^29^, Chinese^30^, Persian^31^, Norwegian^17^, Malaysian^32^, Brazilian^33^, Indonesian^34^, Thai^35^, Arabic^36^, Indian^37^, and Polish^38^ along with linguistic adaptation and psychometric validation.

### Data collection instrument

The study questionnaire consisted of 3 distinct sections including, (a) Socio-demographic questionnaire, (b) Diabetic and general health profile questionnaire and (c) Bangla version of DDS-17 (BDDS-17). The socio-demographic section included personal information such as age, sex, monthly family income, and highest educational attainment (years of schooling), family history of diabetes, age at diagnosis and duration of diabetes. The diabetic and general health profile section included information on diabetic complication, comorbidity, diabetes medication(s), anthropometric measurements, clinical and biochemical reports. Diabetes profile, diagnosis and treatment history and relevant medical information and the latest biochemical reports of the patients were obtained from the patient’s treatment record book. Demographic and other health related information collected through questionnaire were crosschecked with the patient’s treatment record book. The third section included The Bangla version of DDS-17 (BDDS-17), which was developed through translating 17-item English DDS^24^ questionnaire by two professional translators in consensus. Back translation was done by a native English speaker to ensure conceptual and semantic equivalence. Translating and back translating helped streamline the translation cross-culturally. None of the items required modification. The BDDS-17 was pretested in 30 diabetic patients who were not included in the testing of the score. None of the participants reported any difficulty in understanding the questions. The questionnaire took on an average 20 min to complete. Each of the items were scored on a 6-point Likert scale ranging from 1 (less concerned) to 6 (more concerned) based on severity of problem individual is facing in relation to that item^24^. The overall BDDS score is generated by averaging the score of 17 items. The score of each of the 4 domains is generated by averaging scores of the items of the respective domains. For the total score of overall DDSs or any specific domain, a score of < 2 indicating little or no distress; between 2 and 2.9 indicates moderate distress, and score ≥ 3 indicates high distress^18^.

### Statistical analyses

Statistical analyses were performed using statistical software R version 5.5.3 (The R Project for Statistical Computing)^39^. Socio-demographic and disease profile of the participating patients were presented using appropriate descriptive statistics. Factor analysis was conducted to assess cross-loading of the scale domains, using principal component extraction and direct oblimin rotation. An eigenvalue > 1.0 was considered as cut-off for extraction of components^40^. Items were screened for factor loading > 0.5. Published evidence suggests that distress is higher among patients with complications^28,41^ and those are on insulin treatment^42,43^. To test convergent and discriminant validity of the scale total score and the specific domain scores were compared across status of diabetic complications (no complication vs one or more complications) and insulin treatment (on insulin vs not on insulin treatment alone or with oral hypoglycemic agent) using independent t test.

Internal consistency was assessed using Cronbach’s alpha (α) coefficient. The α for 17-item scale and for each of the domains were computed. An α > 0.70 was considered as adequate internal consistency^44^. Item-total correlation and effect of removal the item from the scale on the internal consistency of the scale’s was determined. Item-total correlation coefficient (r) > 0.4 was considered as adequate^45^. The BDDS-17 was re-administered at a 4-week interval on a subset of patients to assess the stability (test–retest reliability). Paired comparison of the global score of the scale and domains across repeated administrations on same individuals were done using paired t-test. Stability were checked using the intraclass correlation coefficient (ICC) with 95% confidence intervals (95% CI). Excellent agreement was considered with ICC ≥ 0.75, good agreement with ICC 0.60–0.74, fair to moderate agreement with ICC 0.40–0.59 and poor agreement was considered with < 0.40^46^. Agreement across two administrations of the questionnaire was assessed by generating weighted Kappa (κ) for the scale and for all four domains.

## Results

### Participant’s characteristics

A total of 1184 patients with T2DM were included in the study. The baseline and demographic profile of participants are presented in Table 1. Average age of the patients was 50.1 ± 12.1 years, 72.2% were female, 46.5% were urban residents, 22.6% had no formal schooling or institutional education. More than three quarters (77.3%) of the patients had a family history of diabetes in first degree relative. Average age at diagnosis of diabetes of the participants 43.8 ± 11.4 years and the duration of diabetes was 7.2 ± 6.0 years and 44.7% patients were on insulin treatment. About half (48.7%) of the patients reported to have one or more diabetic complication (such as diabetic nephropathy, diabetic neuropathy, diabetic retinopathy, diabetic foot and sexual disfunction), while 63.7% had one or more co-morbidities (such as, cancers of lung, stomach, uterus and breast, hypertension, ischemic heart disease, hypo-or-hyperthyroidism, dyslipidemia, osteoporosis, osteoarthritis, asthma, joint pain, bronchitis). Based on the BDDS-17 scale used among the patients, 22.5% had diabetic distress. More specifically, 59.5% had emotional burden, 39.4% had regimen-related distress, 3.8% had physician-related distress and 12.2% had interpersonal distress.

**Table 1:** Characteristics of the participants (n = 1184).

Variables	N (%)
**Age in years**	
< 40 years	208 (17.6)
40–49 years	293 (24.7)
50–59 years	349 (29.5)
≥ 60 years	334 (28.2)
Mean ± SD years	50.1 ± 12.1
**Sex**	
Male	353 (29.8)
Female	831 (70.2)
**Monthly family income**	
< 20,000 taka	239 (20.2)
20,000–39,999 taka	466 (39.4)
40,000–59,999 taka	295 (24.9)
≥ 60,000 taka	184 (15.5)
Mean ± SD taka	39,034 ± 62,641
**Area of residence**	
Metropolitan	551 (46.5)
Rural	633 (53.5)
**Educational attainment**	
No formal education	268 (22.6)
Primary	464 (39.2)
Secondary or equivalent	246 (20.8)
Higher secondary or above	206 (17.3)
**Family history of diabetes**	
Negative	269 (22.7)
Positive	915 (77.3)
**Age at diagnosis of diabetes**	
< 30 years	121 (10.2)
30–39 years	309 (26.1)
40–49 years	385 (32.5)
≥ 50 years	369 (31.2)
Mean ± SD years	43.8 ± 11.4
**Duration of diabetes**	
< 5 years	487 (41.1)
5–9 Years	333 (28.1)
10–14 years	219 (18.5)
≥ 15 years	145 (12.2)
Mean ± SD years	43.8 ± 11.4
**On insulin treatment**	
No	655 (55.3)
Yes	529 (44.7)
Diabetic complication	
None	607 (51.3)
One or more	577 (48.7)
**Major comorbidities**	
None	430 (36.3)
One or more comorbidities	754 (63.7)

### Validity of BDDS-17

#### Construct validity

**Figure 1:**
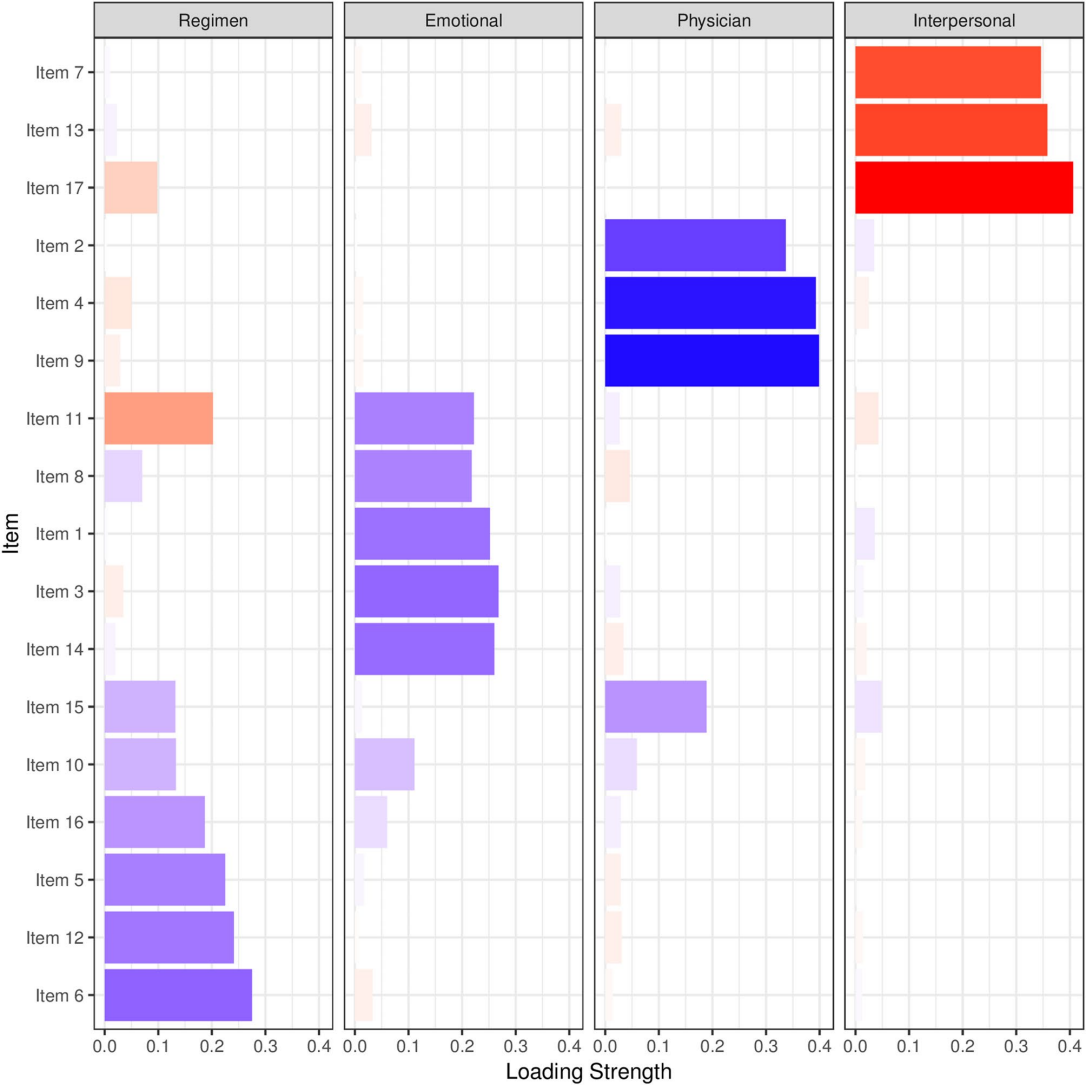
Component loading plot depicting extraction of 4 domains of BDDS-17 [emotional burden (EB), regimen-related distress (RR), physician-related distress (PR), interpersonal distress (IP)]. Items presented in vertical axis and are clustered according to the components (domains), Loading strengths are plotted at the horizontal axis. *Loading gradient high = blue, mid = "white", low = "red", midpoint = 0.

Principal component analysis extracted 4 components similar to the domains of original DDS-17 [emotional burden (EB), regimen-related distress (RR), physician-related distress (PR), interpersonal distress (IP)] (Fig. 1). These components together explain 66.5% of the variance. Only item 15 showed cross loaded in both PR (0.522) and RR (0.537) domains (Supplementary table 1).

#### Internal consistency

Psychometric properties BDDS-17 items and the domains are presented in supplementary table 2 and in Table 2. Cronbach’s α for the scale and for each of the domains were computed. The overall α for the scale was adequately high (0.838) indicating the scale was reliable for capturing the distress among diabetes patients. All items, except items 2 and 11, were correlated with the total score (r > 0.4). Removal of all the items, except item 11, results in reduction of global α values suggesting their attribution to the scale. Only removal of Item 11 results in increase of global α values (0.838 vs 0.859). The internal consistency (α) for the domain’s ranges from 0.698 in the PR domain to 0.878 in the IP domain. All 4 domains were highly correlated with the total score. The domain total correlation coefficient (r) ranged from 0.561 in the RR domain to 0.799 in the PR domain.

**Table 2:** Internal consistancy relaibility of BDDS-17 and its domains (n = 1184).

	Items	Mean score (SD)	Cronbach's alpha	Domain total correlation	
				Coefficient	p value
Total score	1–17	2.33 (0.7)	0.838	–	–
Regimen-related distress domain	5, 6, 10, 12, 16	1.27 (0.6)	0.840	0.561	< 0.001
Emotional burden domain	1, 3, 8, 11, 14	3.3 4(1.4)	0.816	0.690	< 0.001
Physician-related distress domain	2, 4, 9, 15	2.55 (1.3)	0.698	0.779	< 0.001
Interpersonal distress domain	7, 13, 17	1.73 (1.3)	0.878	0.649	< 0.001

Cronbach's Alpha and its 95% CI of the 17-item scale and of each of the 4 domains are generated. Correlation coefficient and p value for the domain total correlation are presented in the right two columns.

#### Convergent and discriminant validity

Table 3 presents the comparison of diabetes distress score (Mean ± SD) across patients with and without complication(s) and among patients having diabetic treatment with or without insulin. Patients with one or more diabetic complications (2.53 ± 0.81) scored significantly higher diabetic distress overall than patients with no complication (2.16 ± 0.71), (p < 0.0001). A similar significant difference is evident across all domains (p < 0.001). Patients on insulin treatment (2.48 ± 0.79) scored significantly higher diabetic distress than patients not on insulin (2.22 ± 0.76), (p < 0.0001). The difference of scores between patients with and without insulin were significant across all domains (p < 0. in the interpersonal 0001 to p = 0.012) except distress domain (p = 0.453).

**Table 3:** Diabetes distress score among patients with or without complications and insulin treatment are assess as a measure to see scale’s discrimination capacity (n = 1184).

	Complication of diabetes			On insulin treatment		
	No	Yes	p value*	No	Yes	p value*
	Mean ± SD	Mean ± SD		Mean ± SD	Mean ± SD	
Total score	2.16 (0.71)	2.53 (0.81)	< 0.001	2.22 (0.76)	2.48 (0.79)	< 0.001
Regimen-related distress domain	3.14 (1.40)	3.54 (1.30)	< 0.001	3.23 (1.43)	3.46 (1.27)	0.004
Emotional burden domain	2.28 (1.20)	2.84 (1.34)	< 0.001	2.30 (1.20)	2.87 (1.35)	< 0.001
Physician-related distress domain	1.20 (0.53)	1.35 (0.65)	< 0.001	1.23 (0.57)	1.32 (0.63)	0.012
Interpersonal distress domain	1.57 (1.04)	1.90 (1.20)	< 0.001	1.71 (1.18)	1.76 (1.07)	0.453

*p values are generated using independent t test.

#### Test–retest reliability

Paired comparison in shows no statistically significant difference across repeated administration of the questionnaire on same individuals (p > 0.353), although EB domain, PR domain and IP domain show slight variation between test and retest reliability (Supplementary Table 3). Test retest reliability assessment in terms of both stability (ICC) and agreement (κ) were presented in Table 4. The scale (Overall ICC 0.941) as well as all the 4 domains individually showed high stability over repeated administration at an interval of 4 weeks. ICC of the domains ranged from 0.855 in PR domain to 0.938 in the RR domain. Agreement across repeated measurement also found to be high in total score κ = 0.584) and in the domains, κ ranged from 0.592 in IP domain to 0.691 in the EB domain.

**Table 4:** Test retest reliability and agreement of the scale across two administrations at 4-weeks interval (n = 201).

	Intraclass correlation (ICC)				Weighted Kappa statistics			
	Coefficient	95% CI		p value	*Κ*	95% CI		p value
Total score	0.941	0.923	0.956	< 0.001	0.584	0.456	0.714	< 0.001
Regimen-related distress domain	0.938	0.918	0.953	< 0.001	0.689	0.642	0.735	< 0.001
Emotional burden domain	0.930	0.908	0.947	< 0.001	0.691	0.592	0.791	< 0.001
Physician-related distress domain	0.855	0.809	0.890	< 0.001	0.634	0.554	0.713	< 0.001
Interpersonal distress domain	0.874	0.834	0.905	< 0.001	0.592	0.552	0.658	< 0.001

Interclass correlation coefficient and Kappa statistics are generated along with the 95% CI and p value for total score ad for all 4 domains independently.

## Discussion

The BDDS-17 demonstrated consistent component (domain) structure, good psychometric property and stability in Bangladeshi diabetic population. The scale was found Factor analysis in Bangladeshi diabetic population extracted four distinct factors that matched the critical content domains proposed by Polonsky et al.^24^ and showed similar internal consistency. In Bangladeshi population, however, item 15 “Feeling that I don't have a doctor who I can see regularly enough about my diabetes” cross loaded in both physician-related (Cronbach’s α = 0.522) and regimen-related (Cronbach’s α = 0.537) domains. This incongruity might be attributed to cultural differences of Bangladeshi patients who quite often are skeptic about whether doctors understood the problem and ended up giving wrong treatment. This is a generalized problem in health care setting in Bangladesh because of overload of patients. Doctor can hardly allocate enough time to establish rapport with the patient that may reflect generalized distrust on the physician and the treatment they provide. Exploratory factor analysis finding suggests that the BDDS-17 measures pragmatic domains of diabetes distress and represent relatively parsimonious dimensions of diabetes distress, which is distinct from classical features of depression^47^. Hence, use of this tool would help those with diabetes to express accurately the level, intensity, and characteristics of specific symptoms they endure.

Internal consistency reliability of the BDDS17 was adequately high (α = 0.84). The finding is consistent across cultural and linguistic adaptations in Thai (α = 0.95)^35^, Polish (α = 0.88)^38^, Saudi (α = 0.87)^36^ population to ranging from Asia Pacific Islander (α = 0.94)^48^, Danish (α = 0.92)^29^, Norwegian (α = 0.92)^17^ Chile (α = 0.74)^49^ and Chinese (α = 0.90)^30^ populations suggesting its wider applicability across populations. In Malay population the tool was found to equally reliable while administered in Malaya (α = 0.94)^32^ and English (α = 0.92)^50^ languages, suggesting wider generalizability and cultural adaptability of DDS17. The high internal consistency reliability coefficients suggest a greater degree of item homogeneity and, in a manner, support the usefulness of the BDDS-17 as a severity measure of diabetes distress. The result of the item-total correlations provided further corroboration for the homogeneity across items and is consistent with finding of studies conducted in other population^17,30^. In agreement with previous literature^17,30,32^, diabetes distress in our study was significantly higher in patients on insulin treatment than those not on insulin, suggesting the ability of the tool to discriminate between those who had greater likelihood of having distress than those with least likelihood.

The assessment of distress in diabetic patients is critical as the condition quite often leads to a diagnosis of psychiatric conditions such as depression or anxiety etc.^47^. The assessment typically focuses on an individual's level of suffering and the potential need for intervention. Often repeated assessment of individual is required in course of management. The determination of test–retest reliability is critical for a measure of distress, especially if such a measure is to be used in treatment outcome research or clinically to evaluate symptom change due to therapy. The 4-week stability based on test–retest reliability coefficients found in this study were high (ICC—0.941) with minimal change in domain scores and supports the stability of the BDDS-17 and is particularly allows applications that require repeated assessments. Although, our ICC is slightly lower than that in the Thai version of DDS-17 (ICC—0.97)^35^. This discrepancy may be due to the fact that the Thai study used a 2-week period between the two measures opposed to our 4 weeks test–retest interval. In the Thai study, shorter duration might have promoted recall of previous administration of the tool^35^.

A number of instruments for detecting or screening DD is reported in the current literature—Questionnaire on Stress in Patients with Diabetes-Revised (QSD-R)^22^, Problem Areas in Diabetes scale (PAID)^20^, ATT39^51^ and the 2-item diabetes distress screening instrument (DDS2)^18^. These tools have been associated with quite a few limitations. Some of the important constructs relevant to DD had not been encompassed adequately in these tools or appeared to be ambiguous, if included. For example, PAID only covered patients’ attitudes toward health professionals. PAID included an item on ‘the idea of goals of diabetes care’ and QSD-R included items about ‘irritability’, these items could often be ambiguous. Ideally of the self-reported tools are preferred to be as brief as possible, and also to be composed of multiple domains (subscales) so that the tool can capture the spectrum of distresses. PAID is relatively brief (20 items) but consists on distinct domains. On the other hand, despite having distinct domains the applicability of both ATT39 (45 items) and QSD-R (39 items) are hindered by sheer size of the instruments. The DDS2 can easily be administered and scored, however, its application beyond screening is yet to be established.

High levels of DD have been significantly associated with poor glycemic control, poor self-care, low diabetes self-efficacy, and poor quality-of-life, even after controlling for clinical depression^52^. Integration of the assessment of diabetes distress using a linguistically validated tool, into the routine diabetes care services, may assist clinician better understand the factor that may be linked with poor management of diabetic^13^. Further, by dint of having distinct subscales, the tool can facilitate detection of domain specific distress, that can guide primary care giver as well as clinicians in deciding and individualized care.

A major strength of our study is the representation of the broader population of patients with T2DM in Bangladesh, which was achieved through including reasonably large sample from multiple centres representing plausible strata among Bangladeshi diabetic patients. One limitation of the study was the adoption of convenience sampling for recruitment of study sample. Inclusion of one rural and three metropolitan centres have the potential to pool a sample not representative of the Bangladeshi T2DM patient population. Further, included patients were those attended regular follow-up at the study hospitals for diabetic care, and data were collected in diabetes outpatient clinics of the study hospitals only, which may limit the generalizability of the findings to wider patient population. Scope of social desirability bias also could not be ruled out as certain section of the participants may respond in a socially desirable manner (example elderly or female patients who were interviewed while accompanied by relatives and carers. Lastly, test–retest reliability was assessed on a small subset of the sample, this have the potential to affect the results of the stability estimate of this tool.

To conclude, current study confirms reliability and validity of the BDDS-17 as a measure of diabetes distress among Bengali speaking T2DM patients in Bangladesh. This psychometrically validated tool is likely to efficiently detect distress in Bangladeshi diabetic patients and shall facilitate estimation of the magnitude of the problem the diabetic patients are enduring. The tool can be used in both clinical and research settings for detecting the diabetes-specific distress. Future research is recommended to further validate the tool on more representative population. Future research should focus on detecting burden of the distress in Bangladeshi patient with a view to formulating effective mitigation options.

## Supplementary Information

Supplementary Information.
