# Supplementary material for: Psychometric validation of diabetes distress scale in Bangladeshi population
Source: Sci Rep. 2022 Jan 12;12:562. doi: 10.1038/s41598-021-04671-0 (PMC8755848; doi:10.1038/s41598-021-04671-0)
Supplement: Supplementary file 1 — Supplementary Information. [file 41598_2021_4671_MOESM1_ESM.docx]

**Supplementary tables**

| Item  number | Domain initial* | Item description | Extracted factors/component | | | |
| --- | --- | --- | --- | --- | --- | --- |
|  |  |  | 1 | 2 | 3 | 4 |
| 6 | RR | Feeling that I am often failing with my diabetes routine. | 0.886 |  |  |  |
| 12 | RR | Feeling that I am not sticking closely enough to a good meal plan. | 0.802 |  |  |  |
| 5 | RR | Feeling that I am not testing my blood sugars frequently enough. | 0.749 |  |  |  |
| 16 | RR | Not feeling motivated to keep up my diabetes self-management. | 0.700 |  |  |  |
| 10 | RR | Not feeling confident in my day-to-day ability to manage diabetes. | 0.573 |  |  |  |
| 14 | EB | Feeling overwhelmed by the demands of living with diabetes. |  | 0.840 |  |  |
| 3 | EB | Feeling angry, scared, and/or depressed when I think about living with diabetes. |  | 0.835 |  |  |
| 1 | EB | Feeling that diabetes is taking up too much of my mental and physical energy every day. |  | 0.781 |  |  |
| 8 | EB | Feeling that diabetes controls my life. |  | 0.722 |  |  |
| 11 | EB | Feeling that I will end up with serious long-term complications, no matter what I do. |  | 0.624 |  |  |
| 9 | PR | Feeling that my doctor doesn't take my concerns seriously enough. |  |  | 0.841 |  |
| 4 | PR | Feeling that my doctor doesn't give me clear enough directions on how to manage my diabetes. |  |  | 0.824 |  |
| 2 | PR | Feeling that my doctor doesn't know enough about diabetes and diabetes care. |  |  | 0.705 |  |
| 15 | PR | Feeling that I don't have a doctor who I can see regularly enough about my diabetes. | 0.537 |  | 0.522 |  |
| 17 | IP | Feeling that friends or family don't give me the emotional support that I would like. |  |  |  | -0.906 |
| 13 | IP | Feeling that friends or family don't appreciate how difficult living with diabetes can be. |  |  |  | -0.896 |
| 7 | IP | Feeling that friends or family are not supportive enough of self-care efforts. |  |  |  | -0.881 |
|  |  | Eigenvalues of Factors/components | 5.357 | 2.705 | 1.723 | 1.535 |
|  |  | % variance explained by factors/ components | 31.5 | 15.9 | 10.1 | 9.0 |

**Supplementary table 1.** Factor loading of 17 item DDS for the extracted factors/components (n=1184).*EB- Emotional Burden; RR- Regimen-related Distress; PR- Physician-related Distress; IP -Interpersonal Distress. *Extraction Method: Principal Component. Rotation Method: Direct oblimin with Kaiser Normalization. Eigenvalue cutoff for component extraction > 1.0

| Items  Number | Item  Mean (SD) | Scale Mean if Item Deleted | Corrected Item-Total Correlation | Cronbach's Alpha if Item Deleted |
| --- | --- | --- | --- | --- |
| DDS 1 | 4.07 (1.7) | 35.63 | 0.477 | 0.827 |
| DDS 2 | 1.15 (0.6) | 38.56 | 0.293 | 0.837 |
| DDS 3 | 3.04 (1.7) | 36.67 | 0.489 | 0.826 |
| DDS 4 | 1.27 (0.9) | 38.43 | 0.333 | 0.835 |
| DDS 5 | 2.62 (1.6) | 37.09 | 0.481 | 0.827 |
| DDS 6 | 3.35 (1.9) | 36.36 | 0.455 | 0.829 |
| DDS 7 | 1.87 (1.3) | 37.84 | 0.556 | 0.824 |
| DDS 8 | 3.28 (1.7) | 36.43 | 0.576 | 0.821 |
| DDS 9 | 1.24 (0.8) | 38.47 | 0.353 | 0.834 |
| DDS 10 | 2.17 (1.6) | 37.54 | 0.630 | 0.818 |
| DDS 11 | 2.92 (2.1) | 36.78 | 0.052 | 0.859 |
| DDS 12 | 2.51 (1.6) | 37.20 | 0.477 | 0.827 |
| DDS 13 | 1.89 (1.3) | 37.82 | 0.529 | 0.825 |
| DDS 14 | 3.36 (1.7) | 36.35 | 0.606 | 0.819 |
| DDS 15 | 1.42 (1.0) | 38.29 | 0.428 | 0.831 |
| DDS 16 | 2.12 (1.6) | 37.59 | 0.580 | 0.821 |
| DDS 17 | 1.44 (1.0) | 38.27 | 0.453 | 0.829 |

**Supplementary table 2.** Psychometric properties of the 17 items of diabetes distress scale. Item

Mean (SD) and total scale mean if the Item is deleted are generated on 1184 respondent data. Item-total correlation are presented as correlation efficient (r). Cronbach's Alpha of the total scale is generated excluding the item from the scale to show its contribution to the total scale.

| Pairs | Before – after differences | | t | P value |
| --- | --- | --- | --- | --- |
|  | Mean | SD |  |  |
| Item 1 | 0.21 | 1.16 | 2.61 | 0.010 |
| Item 2 | -0.02 | 0.42 | -0.67 | 0.506 |
| Item 3 | 0.18 | 0.91 | 2.81 | 0.005 |
| Item 4 | 0.01 | 0.47 | 0.30 | 0.764 |
| Item 5 | 0.04 | 0.95 | 0.52 | 0.602 |
| Item 6 | -0.06 | 0.99 | -0.85 | 0.395 |
| Item 7 | -0.13 | 0.81 | -2.35 | 0.020 |
| Item 8 | 0.04 | 0.98 | 0.51 | 0.614 |
| Item 9 | -0.01 | 0.50 | -0.28 | 0.778 |
| Item 10 | 0.12 | 0.95 | 1.79 | 0.075 |
| Item 11 | 0.00 | 0.47 | 0.00 | 1.000 |
| Item 12 | -0.07 | 0.98 | -0.94 | 0.351 |
| Item 13 | -0.17 | 0.64 | -3.88 | 0.000 |
| Item 14 | 0.01 | 1.13 | 0.06 | 0.950 |
| Item 15 | -0.06 | 0.74 | -1.04 | 0.298 |
| Item 16 | -0.34 | 1.05 | -4.65 | 0.000 |
| Item 17 | -0.16 | 0.68 | -3.31 | 0.001 |
| EM Domain | 0.09 | 0.54 | 2.27 | 0.024 |
| PR Domain | -0.02 | 0.35 | -0.76 | 0.448 |
| RR Domain | -0.06 | 0.58 | -1.54 | 0.125 |
| IP Domain | -0.16 | 0.50 | -4.39 | 0.000 |
| Total score | -0.02 | 0.36 | -0.93 | 0.353 |

**Supplementary table 3.** Paired difference of 17 items, 4 domains and the total score across two administrations. Pa values are generated using paired t test.
